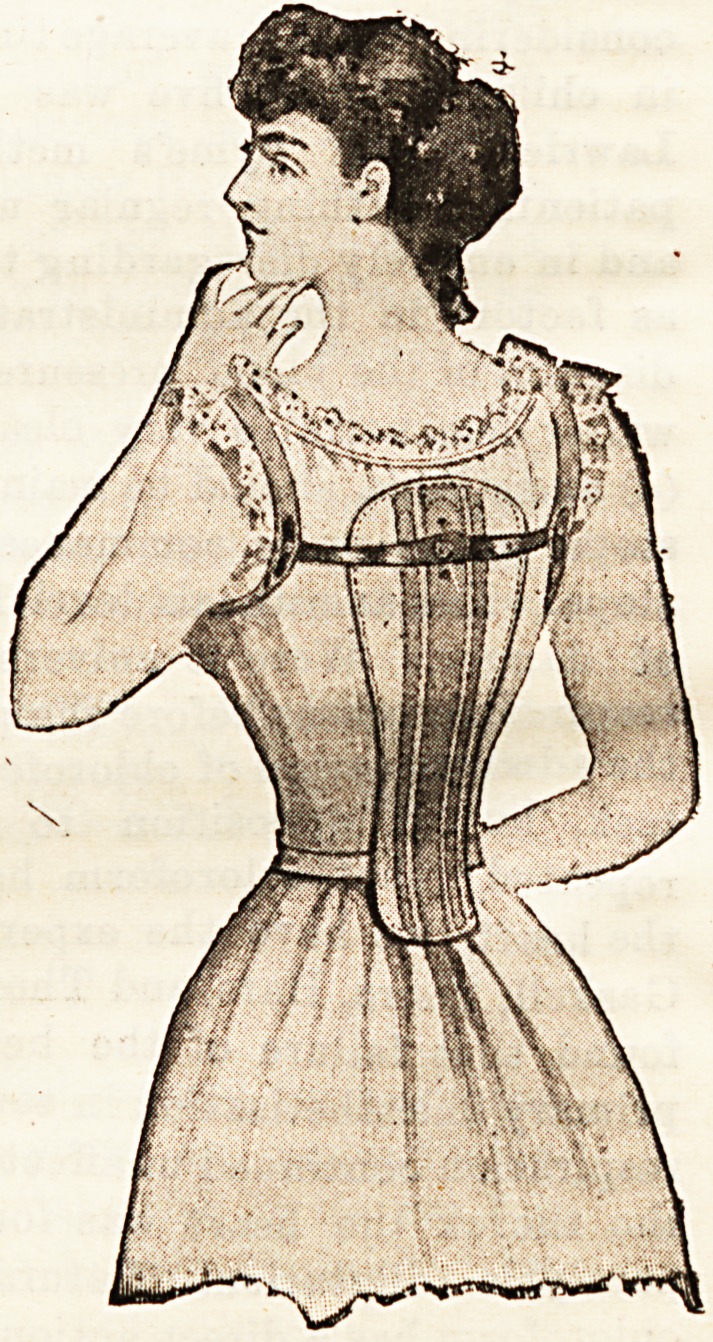# New Appliances and Things Medical

**Published:** 1898-09-24

**Authors:** 


### NEW APPLIANCES AND THINGS MEDICAL.

[We shall be glad to receive, at our Office, 28 & 29, Southampton Street, Strand, London, W.C., from the manufacturers, specimens of all new preparations and appliances which may be brought out from time to time.]

### NEW COMBINED TONGUE DEPRESSOR AND THERMOMETER CASE.

(Lynch and Co., Limited, 192, Aldersgate Street, E.C.)

Dr. T. B. Gabe, of Swansea, is responsible for the idea which resulted in this useful combination of two of the most essential articles of a physician's equipment—namely, the thermometer and the tongue depressor. By holding the tongue depressor, the thermometer index may be easily shaken down if the thermometer is placed bulb downwards in the case. This contrivance should be found most useful to the over-loaded practitioner, and an economy in space and money certainly results.

### HANSEN'S OATMEAL COCOA.

(The Servus Co., 65 and 66, Basinghall Street, London, E.C.)

This is a useful combination of the alkaloid or stimulating properties of cocoa with the nutritive and flesh-forming constituents of a cereal. Tea, coffee, or cocoa as such are eminently unsuited for children. Combined with large quantities of milk they may constitute foods in the ordinary sense, but even then hardly adapted for children. Hansen's oatmeal cocoa is a real food, combined with slight stimulating properties. For young children with a tendency to rickets or scrofula it is highly indicated—as a rule, it is very much to their taste, and will be found economical and simple to prepare in the nursery.

### DOMEN STOOP-CURE.

(Domen Belt Co., 456, Strand, W.C.)

This stoop-cure is constructed of a watch-spring back piece shaped to and supporting the spine. From its centre two lateral springs arise, passing behind the shoulders, and fixed to them by straps which encircle the top of the arm. These springs exercise a backward pull on the shoulders, and the spine piece distributes the pressure along the vertebral column in a uniform and regular manner. In cases where artificial support to the spine and passive pressure to keep the shoulders back are considered advisable, the Domen stoop-cure seems a comfortable belt, and adapted to this purpose. In most cases of round shoulders and spinal curvature these conditions are due to weak and flabby muscles, and cured best by giving the muscles more to do by properly graduated exercises.

## Figures and Tables

**Figure f1:**
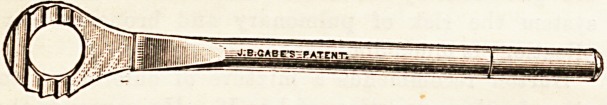


**Figure f2:**